# The D-lactate enigma: exploring the inflammatory influence of D-lactate in cattle

**DOI:** 10.3389/fvets.2024.1509399

**Published:** 2024-12-18

**Authors:** Rafael Agustín Burgos, Carolina Manosalva, Pablo Alarcón, Max Navarro, John Quiroga, Gabriel Morán, Jan Gallastegui, Sebastián Brauchi, María Daniella Carretta

**Affiliations:** ^1^Laboratory of Inflammation Pharmacology and Immunometabolism, Institute of Pharmacology and Morphophysiology, Faculty of Veterinary Sciences, Universidad Austral de Chile, Valdivia, Chile; ^2^Institute of Pharmacy, Faculty of Sciences, Universidad Austral de Chile, Valdivia, Chile; ^3^Institute of Veterinary Clinical Sciences, Faculty of Veterinary Sciences, Universidad Austral de Chile, Valdivia, Chile; ^4^Institute of Physiology, Faculty of Medicine, Universidad Austral de Chile, Valdivia, Chile

**Keywords:** bovine, D-lactate, inflammation, ruminal acidosis, lameness

## Abstract

D-lactic acidosis is associated with fermentative disturbances and is often marked by elevated levels of D-lactic acid in the blood, ruminal fluid, and synovial fluid in cattle. D-lactic acidosis is linked to various inflammatory manifestations, and although the causative factors have been extensively explored, the exact pathogenesis of the associated inflammation remains elusive. Notably, less attention has been given to D-lactate, a stereoisomer found in the plasma of affected animals, which may lead to D-lactic acidosis. This review aims to highlight the evidence suggesting that D-lactate participates in the modulation of inflammatory processes and explore its potential effects on synoviocytes, polymorphonuclear neutrophils, macrophages, and T-cells. This comprehensive examination of D-lactate’s involvement in the inflammatory response process provides timely insights into the pathophysiological aspects of ruminal acidosis in cattle.

## 1 Introduction

Lactate exists as two stereoisomers, L-lactate and D-lactate, both of which play distinct roles in metabolism. L-lactate is the predominant form in human metabolism, is produced primarily during anaerobic glycolysis, and serves as a key intermediate in cellular energy homeostasis (1). In contrast, D-lactate, though less abundant, is produced by specific bacteria and has been associated with certain pathological conditions, such as D-lactic acidosis, particularly in patients with short bowel syndrome or gut dysbiosis (1–3).

Despite their structural similarities, these two isomers are metabolized through distinct enzymatic pathways. L-lactate is processed predominantly in the liver and heart through L-lactate dehydrogenase (LDH), whereas D-lactate metabolism occurs via D-2-hydroxyacid dehydrogenase, an enzyme present in fewer tissues with a different regulatory mechanism (2, 4, 5). This distinction is crucial since the accumulation of D-lactate can have neurotoxic effects, leading to symptoms such as confusion and ataxia (6).

Once exclusively considered a metabolic waste by-product, lactic acid is now acknowledged as a pleiotropic signal involved in diverse physiological and pathological conditions (1). Lactic acid is present as a lactate-conjugated base at a physiological pH of 7.4, and exists in two enantiomeric forms in mammals: L-lactate is produced during glycolysis when oxygen levels are low (3), while D-lactate is formed through the detoxification of methylglyoxal (2).

L-lactate is a naturally occurring enantiomer in mammals (4). In cattle blood, the normal range of L-lactate is 0.5–1 mM (7). In contrast, the D-lactate levels are in the micromolar ranges (8). Elevated L-lactate levels beyond this physiological range lead to hyperlactatemia, which is often triggered by various pathological processes are associated with deficient mitochondrial adenosine triphosphate (ATP) production (e.g., hypoxia), increased aerobic glycolysis (e.g., the Warburg effect, diabetic acidosis), or decreased lactate removal (9). Unlike hyperlactatemia, D-lactate accumulation, known as D-lactic acidosis, is observed in cases of ethylene glycol or propylene glycol poisoning (10). Dissimilar to L-lactate, D-lactate produced by alcohol dehydrogenase in the liver is not effectively metabolized by aldehyde dehydrogenases, leading to D-lactate accumulation (9, 11, 12). In humans with diabetic ketoacidosis, plasma D-lactate levels are significantly higher, reaching 3.44 ± 1.99 mM, compared to the 0.48 ± 0.56 mM in patients with diabetes without ketoacidosis (13, 14). D-Lactic acidosis can develop as a complication after surgical procedures such as small bowel resection, which is associated with short bowel syndrome and intestinal bypass surgery performed for the treatment of obesity (15, 16). The clinical manifestation of short bowel syndrome includes episodes of metabolic encephalopathy, similar to those observed in cases of ethanol intoxication (15).

The etiology of D-lactic acidosis in bovines is often due to disruptions in digestive fermentation caused by excessive grain and carbohydrate intake, or infectious diarrhea, commonly observed in neonatal calves (2, 7, 17). In addition, D-lactic acidosis in calves can be caused by a malfunction of the esophageal groove reflex, resulting in the diversion of milk into the reticulorumen (ruminal drinking) instead of direct delivery to the abomasum (18). The consequences of D-lactic acidosis in cattle have been described previously (7, 19). However, the role of D-lactate in the pathophysiology of associated diseases has not been the focus of attention. This review focuses on new advances in understanding D-lactate as a potential metabolic indicator associated with cellular damage.

Although the role of L-lactate in exercise, hypoxia, and metabolic diseases has been extensively studied, the physiological and pathophysiological roles of D-lactate remain poorly understood. Additionally, the clinical significance of D-lactate and its potential contributions to metabolic disorders beyond the known cases of D-lactic acidosis are still emerging research areas. This review aims to clarify these gaps by synthesizing current knowledge on the distinct roles and metabolic pathways of L-and D-lactate, focusing on the underexplored implications of D-lactate in health and disease.

## 2 Biochemistry and the metabolism of D-lactate

**Figure 1:**
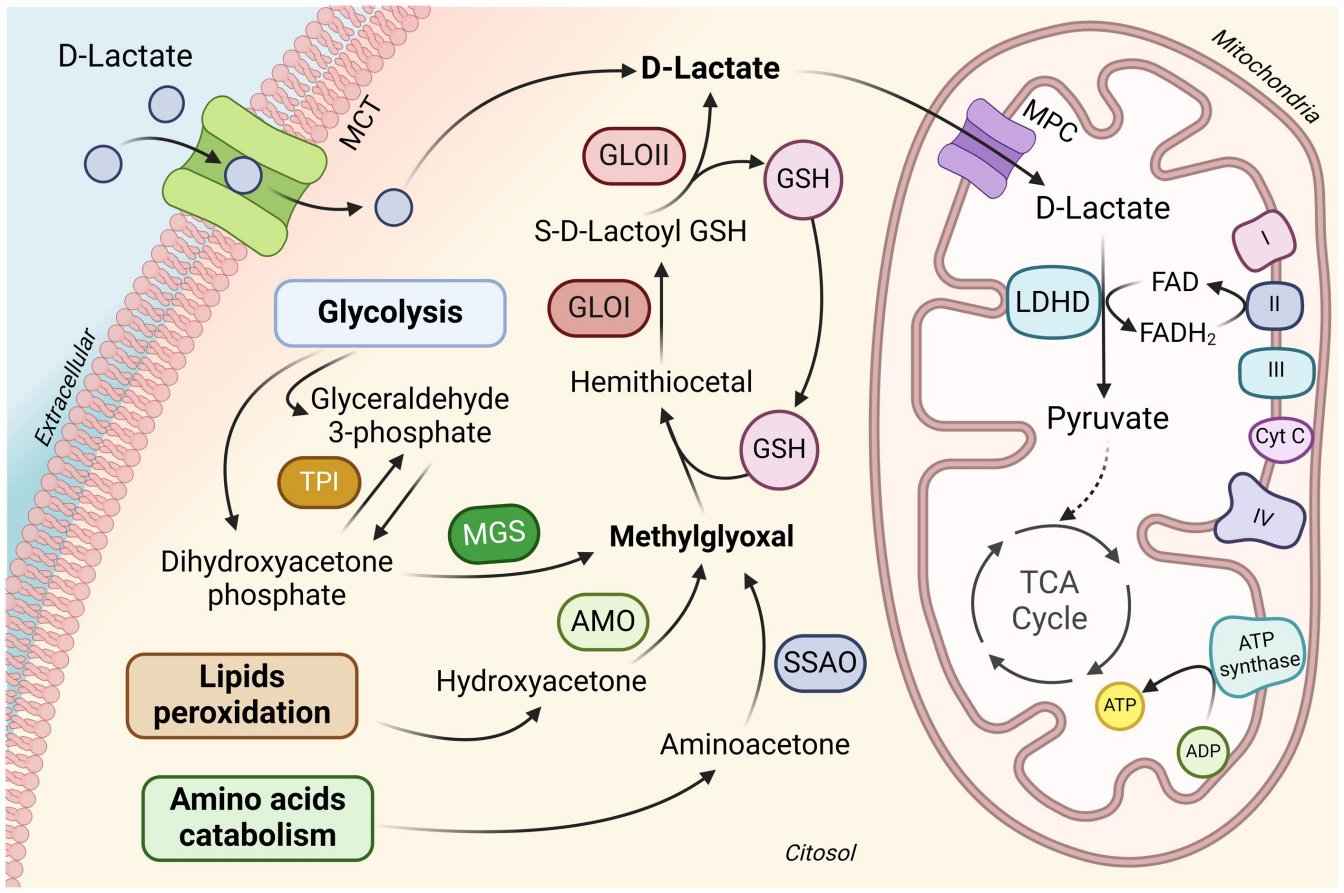
D-lactate metabolism in mammalian cells. D-lactate enters from the extracellular into the cytosol via MCT. Mammalian cells can also generate D-lactate during methylglyoxal metabolism. Methylglyoxal is mainly produced as a byproduct of glycolysis, although it can also be produced during lipid peroxidation and amino acid breakdown. Detoxification of methylglyoxal takes place in the cytosol by the glyoxalase system, consisting of the enzymes GLO1 and GLO2, which employ reduced GSH to form D-lactate. D-lactate metabolism occurs within the mitochondria, entering through MPC located in the inner mitochondrial membrane. Within the mitochondria, D-lactate is oxidized to pyruvate by LDHD, using FAD as a cofactor. Pyruvate continues its oxidation to enter the mitochondrial tricarboxylic acid cycle. MCT, monocarboxylate transporter; TPI, triose phosphate isomerase; MGS, methylglyoxal synthase; AMO, acetol monooxygenase; SSAO, semicarbazide-sensitive amine oxidase; GSH, glutathione; GLOI, glyoxalase 1; GLOII, glyoxalase II; MPC, mitochondrial pyruvate carrier; LDHD, D-lactate dehydrogenase; FAD/FADH_2_, flavin adenine dinucleotide; TCA, tricarboxylic acid. Figure created with BioRender.

In mammals, D-lactate is produced through the breakdown of carbohydrates and lipids during the generation of methylglyoxal (MG) (Figure 1). MG is a byproduct of glycolysis and is formed by the fragmentation of dihydroxyacetone phosphate (DHAP) and glyceraldehyde 3-phosphate (G3P) (20). Triose phosphate isomerase metabolizes G3P into DHAP, and the conversion of DHAP to MG is catalyzed by methylglyoxal synthase (MS) (21). Moreover, MG can be formed by protein catabolism, which includes the synthesis of aminoacetone by aminoacetone synthetases, and from lipid metabolism via reactions facilitated by glycerol kinase and glycerol-3-phosphate dehydrogenase in both the kidneys and the liver. These processes connect glycolysis and lipid metabolism during MG production (22). The primary detoxification pathway for methylglyoxal is the ubiquitous glyoxalase system (23). This system consists of two enzymes, glyoxalase I (GLO1) and glyoxalase II (GLO2) and utilizes a catalytic amount of reduced glutathione (GSH) to form D-lactate (24).

In cattle, the shift from forage-based diets to concentrate-rich diets, which contain higher amounts of rapidly fermentable carbohydrates (such as during lactation or feedlot rearing), significantly alters the rumen fermentation process. The sudden intake of these carbohydrates, for example, due to sorting behavior, further drives the fermentation process to focus more on non-fibrous carbohydrates (25). In the rumen, these carbohydrates are rapidly fermented into short-chain fatty acids (SCFA) and lactate, leading to the accumulation of these acids and a decrease in ruminal pH below normal fermentation levels (i.e., pH < 5.6) (26). Here, D-Lactate is produced by the gut microbiota through lactate racemases, which are nickel-dependent enzymes present in halophilic archaea, such as *Haloarcula marismortui* and also in several commensal bacteria, including *Lactobacillus* and *Clostridium* (27). Many bacteria in the rumen, such as *Streptococcus bovis* and *Lactobacillus*, produce D-lactate (19, 28, 29). However, other microorganisms within this digestive environment, including *Megasphaera elsdenii (M. elsdenii)*, *Selenomonas ruminantium* subsp. *Lactilytica,* and certain entodiniomorph protozoa, also utilize lactate (1, 30). Approximately 60–80% of DL-lactate fermentation in the rumen is attributed to *M. elsdenii* (28). Lactate-utilizing microorganisms are sensitive to a decrease in pH, which promotes the proliferation of lactate-producing bacteria and consequently induces ruminal D-lactic acidosis (19, 26).

In pre-weaning calves with neonatal diarrhea due to viral infections or *Cryptosporidium*, the prevalence of D-lactic acidosis seems to be linked to villous atrophy in the small intestine (31). This condition can lead to impaired digestion and absorption, subsequently fostering microbial fermentation of substrates in the large intestine (6, 31). In humans, D-lactic acidosis is frequently recognized as an outcome of short bowel syndrome, often arising after surgical intervention involving partial removal of the small intestine due to malignant tumors, diseases, or procedures such as jejunoileal bypass surgery (32, 33). A reduction in the length of the small intestine compromises the carbohydrate absorption capacity, leading to an elevated carbohydrate flow to bacteria in the colon. This increased influx may promote bacterial proliferation, creating an acidic environment that supports D-lactate production (32, 33).

Varying rates of D-lactate oxidation have been observed in bovine tissue analyses (34), with the highest oxidation rates observed in the kidney cortex, followed by the heart and liver, and muscle tissue displaying the lowest oxidation rates. Similarly, L-lactate oxidation was notably higher in the kidney cortex and heart, with the liver and muscle exhibiting similar rates. Notably, gluconeogenesis oxidation rates for D-and L-lactate are comparable at 0.1 mM lactate concentrations in kidney and liver. However, the proportional utilization of D-lactate relative to L-lactate declined as substrate concentrations increased. These findings emphasize the constrained capacity for D-lactate utilization by bovine tissues (35).

D-lactate metabolism is attributed to oxidation to pyruvate by D-Lactate dehydrogenase (LDHD) (36, 37). Two distinct categories of LDHDs exist: nicotinamide adenine dinucleotide (NAD)-dependent LDHDs and flavin adenine dinucleotide (FAD)-dependent LDHDs (38, 39). The NAD-dependent LDHDs, identified within certain bacterial strains, are classified within the D-isomer specific 2-hydroxyacid dehydrogenase superfamily. Nonetheless, the exact biological roles of these enzymes within bacterial systems remain elusive (40, 41). The FAD-dependent LDHDs were first identified in bacteria, archaea, yeasts, and plants (42–45). These enzymes do not share a common ancestry with either L-lactate dehydrogenases or NAD-dependent LDHDs. They are categorized within the 2-hydroxyacid dehydrogenase subfamily of the vanillyl alcohol oxidase/para-cresol methylhydroxylase (VAO/PCMH) flavoprotein family. All members of this subfamily share a conserved FAD-binding domain and a variable substrate-binding domain. Unlike its NAD-dependent counterparts, FAD is used as a cofactor instead of NAD (38, 39, 46). The FAD-dependent LDHDs have been identified in mammals, including humans, mice, and bovine (36, 38), exhibiting significant sequence resemblance to yeast LDHDs (36, 47, 48). This LDHD demonstrates elevated levels of expression in tissues characterized by high metabolic rates and abundant mitochondria (49), mostly located in the liver and kidney (50) and situated in the inner membrane of mitochondria (36, 47, 48).

## 3 D-lactate absorption

The solute carrier family 16 (SLC16) consists of 14 members categorized within the monocarboxylate transporter (MCT) family (51). These transmembrane proteins play a crucial role in facilitating the transport of lactate enantiomers and various other metabolically essential monocarboxylates, such as pyruvate, branched-chain oxoacids, SCFA, and ketone bodies, across cellular membranes (51, 52). The MCT1-4 group are proton-dependent transporters closely associated with the movement of by-products of the glycolysis cycle across the plasma membrane. These include lactate, pyruvate, and ketone bodies such as acetoacetate and *β*-hydroxybutyrate (51). The *K*_m_ has been reported at 27.5 mM for D-lactate and 4.54 mM for L-lactate in Ehrlich-Lettre Tumor Cells (53). Furthermore, the uptake of D-lactate by MCT-1 is significantly lower than that of L-lactate, with both isomers displaying reciprocal inhibitory effects (54).

The absorption of L-lactate in the digestive tract of monogastric is through the MCT-1, that is localized in the apical membrane and basolateral membrane of the intestinal epithelium (55, 56). In ruminants, MCT1-4 are mostly expressed in the forestomach and large intestine epithelia (57–60). MCT-1 and MCT-2 are primarily localized in the basolateral membrane, with MCT-4 showing a slightly greater orientation towards the luminal side of the rumen epithelia (57, 59, 60). Therefore, lactate anions can enter the ruminal epithelial cells during cotransport with their protons (61). During ruminal acidosis, the concentrations of D-lactate in ruminal fluid reach 50–80 mM (7, 62, 63), suggesting that this condition may be key for MCTs D-lactate absorption. However, in contrast to SCFA, it has been suggested that lactate is more slowly absorbed (64). This could be involved to the higher *K*_m_ of D-lactate (519 mM) than L-lactate (28 mM) observed with MCT-4 (65). Despite this, a decrease in ruminal pH due to excessive SCFA production promotes increased D-lactate accumulation through microbiome modifications (19, 26). In addition, has been reported that an acidic environment reported in ruminal acidosis can decrease the *K*_m_ of MCT-4 for lactate (66). Moreover, the drop in ruminal pH facilitates the passive transport of lactate in its unprotonated form (lactate ion) through the ruminal wall, since the pKa of lactate is 3.84. At this pH, a significant portion of lactate remains unprotonated, allowing it to easily cross the ruminal wall. This process contributes to the development of metabolic acidosis in cattle.

Lactic acidosis becomes evident when plasma L-lactate concentrations exceed 4 mM, potentially resulting in a drop in blood pH to <7.35. Hyperlactatemia, whether mild or severe (i.e., progressing to lactic acidosis), can arise due to multiple factors such as sepsis, hemorrhagic shock, cardiac arrest, trauma, toxic exposure, ischemia, burns, diabetic ketoacidosis, certain types of cancer, and strenuous muscle activity (67). In bovines with ruminal acidosis, the blood lactate is >4 mmol/L after 24 h of ingestion of carbohydrates (7, 62), this becomes even more severe in calves, with concentrations exceeding 10 mM (31).

## 4 D-lactate as a potential proinflammatory agent

During ruminal acidosis, several inflammatory conditions associated with lameness are observed (68, 69). Laminitis is considered the main lesion in cattle with D-lactic acidosis, however, its etiology is still debated (19, 70). Intraruminal injection of lactic acid can induce laminitis in lambs (71, 72). Interestingly, arthritis and concurrent systemic conditions are observed in cattle with laminitis, suggesting a more diffuse inflammatory reaction (73). In fact, the experimental induction of laminitis associated with ruminal acidosis after oligofructose overload leads to an increase in the diameter of the tarsocrural joints (68, 69). Moreover, the concentration of polymorphonuclear leucocytes (PMNs) in the synovial fluid of several joints increases 24 h after induction of carbohydrate overload (68, 74). Additionally, 6 mM of D-lactate and an increase in pro-inflammatory molecules such as Interleukin (IL)-6, IL-1, prostaglandin E (PGE2), and matrix metalloproteinases (MMP)-9 were detected in the synovial fluid of affected bovines (74, 75). Elevated levels of lactate have also been identified in the synovial fluid of humans with various joint diseases such as septic arthritis (76, 77), osteoarthritis (78), osteonecrosis (79), rheumatoid arthritis (RA), and gout (80, 81). Particularly, fibroblast-like synoviocytes (FLS) in RA-affected joints exhibit an increased lactic acid production (82). This increased lactic acid concentration has been proposed to play a pivotal role in the intracellular signaling pathways regulating the production of pro-inflammatory cytokines and is considered a new potential therapeutic target (83–85). D-lactate can modulate the inflammatory response through different cells and mechanisms, contributing to the complex pathophysiology of inflammation observed in cattle. This section explores the distinct effects of D-lactate on fibroblast-like synoviocytes (FLS), polymorphonuclear cells (PMNs), macrophages, and T-cells, highlighting the specific pathways and responses involved in the inflammatory process.

### 4.1 Bovine fibroblast-like synoviocytes

**Figure 2:**
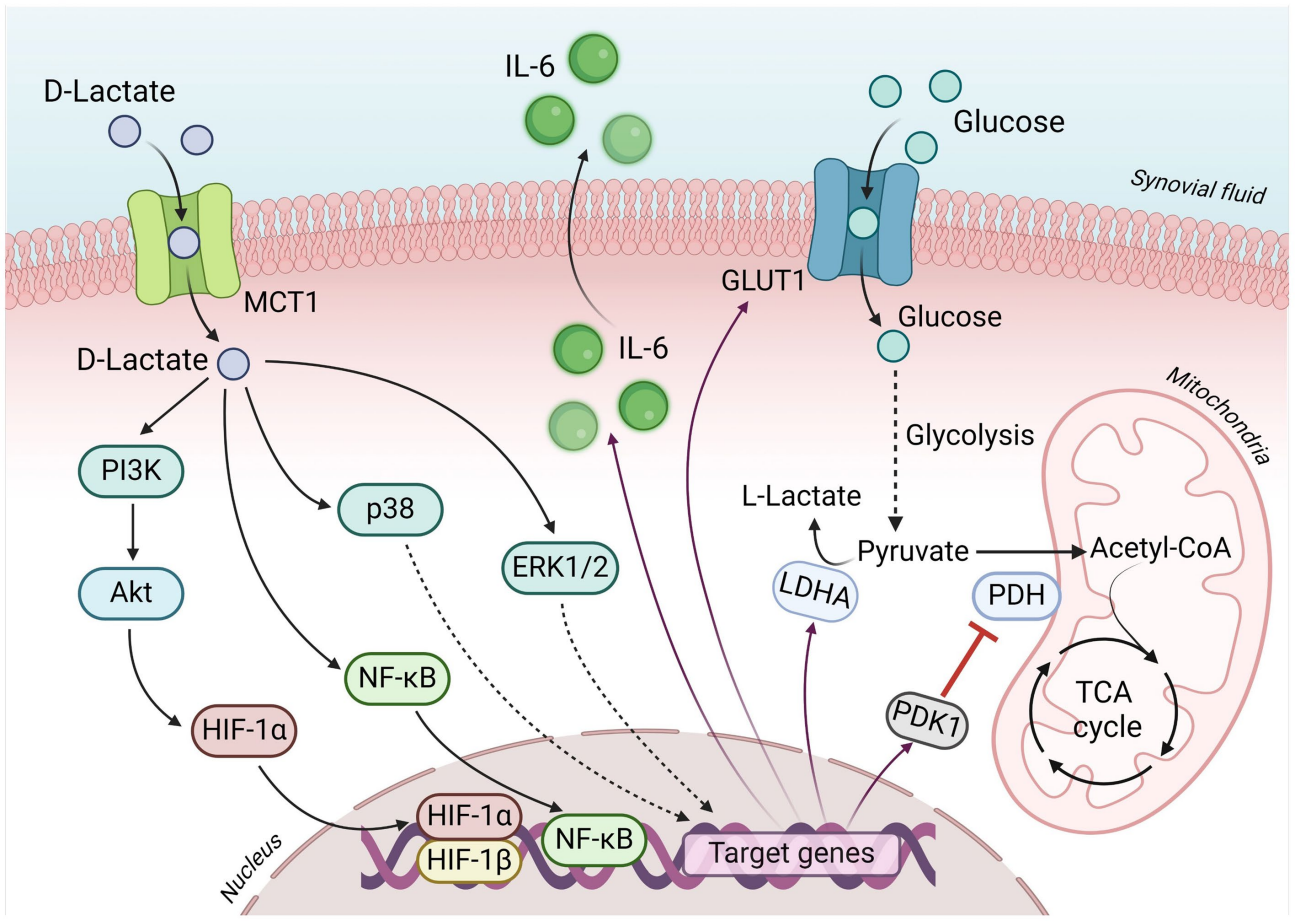
Key signaling events underlying D-lactate-induced metabolic reprogramming in bFLS. D-lactate enters bovine fibroblast-like synoviocytes (bFLS) through MCT-1 and induces the activation of the PI3K/Akt, p38 MAPK and ERK1/2 MAPK pathways, and the subsequent activation of the transcription factors NF-κB and HIF-1. Through these signaling pathways and transcription factors, D-lactate induces the synthesis and secretion of IL-6, an inflammatory marker characteristic of bovine polysynovitis associated with D-lactic acidosis. Additionally, the local inflammatory response is sustained thanks to the activation of HIF-1, which favors glycolytic metabolism by increasing the expression of GLUT1 (which increases the incorporation of glucose from the extracellular medium), PDK1 (blocking the mitochondrial use of pyruvate through the TCA cycle) and LDHA (ensuring glycolytic flux by favoring the oxidation of pyruvate to lactate). IL-6, interleukin 6; MCT1, monocarboxylate transporter 1; GLUT1, solute carrier family 2 (facilitated glucose transporter) member 1; PI3K, phosphatidyl inositol 3-kinase; Akt, protein kinase B; p38, p38 mitogen-activated protein kinase (MAPK); ERK1/2, extracellular signal-regulated kinase 1/2 MAPK; HIF-1α and HIF-1β, hypoxia inducible factor 1 alpha and beta subunits, respectively; NF-κB, nuclear factor kappa B; LDHA, lactate dehydrogenase A subunit; PDK1, pyruvate dehydrogenase kinase 1; PDH, pyruvate dehydrogenase; TCA, tricarboxylic acid. Figure created with BioRender.

Fibroblast-like synoviocytes (FLS) are critical to structure the intimal lining cellular layer of the synovium in diarthrodial joints and are responsible for regulating the composition of the synovial fluid and the extracellular matrix (86). The FLS also define and maintain the inflammatory environment during most arthropathies (87–89). Several antecedents suggest that D-lactate induces specific inflammatory responses in FLS. It has been shown that 5 mM D-lactate or sodium D-lactate induces the expression and production of IL-6 and IL-8 in bovine FLS, suggesting a potential role of D-lactate in joint inflammation during acute ruminal acidosis (90). Additionally, bFLS expresses mostly MCT-1 and its pharmacological inhibition with AR-C155858 was shown to significantly reduce IL-6 and IL-8 secretion induced by D-lactate (90). Furthermore, D-lactate induces the phosphorylation of p38 mitogen-activated protein kinase (MAPK), the extracellular signal-regulated kinase (ERK) 1/2 MAPK, Akt, and triggers the nuclear factor (NF)-κB pathway, which induces cytokine production in bFLS (90). D-lactate can also induce the accumulation of Hypoxia-inducible factor (HIF)-1α and in turn, the expression of IL-6. Moreover, the HIF-1 is closely linked with the PI3K/Akt and NF-κB pathway activation in bFLS (91) (Figure 2).

L-lactate concentrations ranging from 10 to 40 mM have been detected in inflamed tissues of animals with diseases such as arthritis including synovial tissue (92). The RA-FLS express MCT-1 and MCT-4, which regulate lactate uptake and release from cells, respectively (93). An increase in glycolysis (thus lactate levels) has been detected in RA-FLS and is associated with inflammation (94, 95). Moreover, L-lactate can induce an elevation in glycolysis and enhance the glycolytic capacity within synovial fibroblasts (96). Additionally, L-lactate potentiates the tumor necrosis factor-alpha (TNF-*α*)-induced secretion of IL-6 in RA-FLS, indicating a potential activation of the HIF-1 pathway (96). Similarly, D-lactate can also increase the glycolysis augmenting glucose uptake and GLUT-1 expression in bFLS via HIF-1, suggesting a metabolic reprogramming associated with the inflammatory response (91). Moreover, this effect is additionally supported not only by an upregulation of glycolytic enzymes such as LDHA, but also by an upregulation of pyruvate dehydrogenase kinase (PDK)-1, which inhibits Pyruvate dehydrogenase (PDH), hindering the conversion of pyruvate to Acetyl-CoA, ultimately favoring the production of lactate (91) (Figure 1). A PDK-1 inhibitor, such as dichloroacetic acid (DCA), has been utilized to correct the pyruvate influx into the mitochondria, consequently enhancing oxidative phosphorylation and reducing lactate accumulation (97). Studies thus suggest the effectiveness of a PDK-1 inhibitor in reducing inflammation in collagen II-induced arthritis in female DBA/1 mice (98). In bFLS, in which TNF-α increases glycolysis and reduces pyruvate influx into the tricarboxylic acid (TCA) cycle and increasing IL-6 production, DCA effectively reduces the secretion of IL-6 (99). Additionally, metabolic disturbances characterized by increased levels of pyruvate, D-lactate, L-lactate and IL-6 have been detected in the synovial fluid of heifers with acute ruminal acidosis induced by oligofructose overload (75). Thus, the utilization of modifiers targeting glycolytic metabolism emerges as a novel strategy to treat joint inflammation (83–85, 100). This approach warrants attention and exploration within the context of livestock health, especially towards the prevention and management of metabolic-inflammatory diseases in cattle herds.

### 4.2 Polymorphonuclear leucocytes

**Figure 3:**
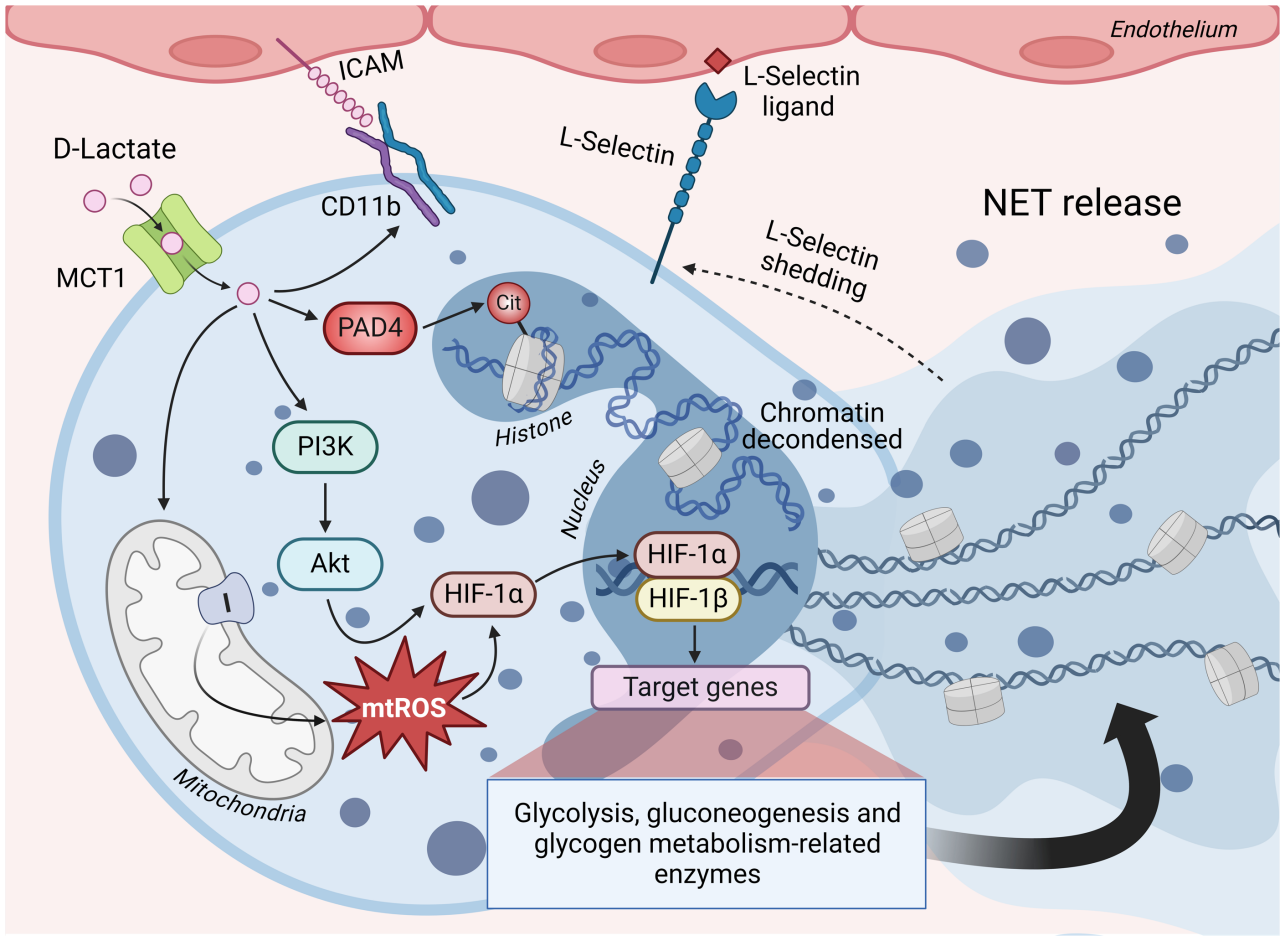
D-lactate-induced NET formation is sustained through metabolic reprogramming of bPMN. Bovine polymorphonuclear neutrophils (bPMN) incorporate D-lactate through MCT-1, which induces the activation of the PI3K/Akt signaling pathway and the subsequent stabilization of HIF-1α. Additionally, D-lactate triggers the activation of PAD4, which catalyzes the transformation of arginine residues to citrulline in histones, leading to chromatin decondensation. HIF-1 activation is primarily responsible for the metabolic reprogramming necessary to energetically sustain NET release, through the overexpression of enzymes associated with glycolysis, gluconeogenesis and glycogen metabolism. D-lactate also increases the production of mtROS, mainly through mitochondrial complex I, a molecular mechanism necessary to induce the stabilization of HIF-1α and, consequently, the greater transcriptional activity of HIF-1. Furthermore, D-lactate promotes bPMN adhesion to endothelial cells through a mechanism involving the increased expression of CD11b and shedding of L-selectin. MCT1, monocarboxylate transporter 1; PI3K, phosphatidylinositol 3-kinase; Akt, protein kinase B; HIF-1α and HIF-1β, hypoxia inducible factor 1 alpha and beta subunits, respectively; mtROS, mitochondrial reactive oxygen species; PAD4, peptidyl arginine deiminase 4; Cit, citrulline; ICAM, intercellular adhesion molecule; NET, neutrophil extracellular traps. Figure created with BioRender.

Polymorphonuclear leucocytes (PMNs) constitute the primary and most abundant leukocyte population in cattle joints 24 h after induced ruminal acidosis (68, 74). PMNs perform various functions, including phagocytosis, generating reactive oxygen species (ROS), and forming neutrophil extracellular traps (NETs) (101, 102). The presence of NETs in synovial fluid is a characteristic of aseptic polysynovitis in cattle (74). While NETs are acknowledged as a beneficial microbicidal mechanism, they are also pathological biomarkers of early rheumatoid arthritis (103, 104). NETs serve as a source of citrullinated autoantigens, significantly amplifying the inflammatory response and triggering the production of inflammatory molecules such as IL-6, IL-8, and adhesion proteins (104). Bovine PMNs (bPMNs) exhibit an induction of NET release upon exposure to D-lactate *in vitro*, a process reliant on its cellular uptake facilitated by MCT-1 transporters, subsequently activating peptidyl arginine deiminase 4 (PAD-4) that induce the histone H4 citrullination (105). Moreover, the release of NET induced PMNs adhesion to bovine endothelial cells by increasing CD11b expression and L-selectin shedding (105) (Figure 3). Similarly, L-lactate has shown the capability to induce NET formation in human neutrophils. Inhibition of lactate dehydrogenase (LDH) activity markedly reduced NETosis induced by PMA and A23187, two well-described NADPH oxidase-dependent and-independent NETosis-inducing stimuli, respectively (106). PMA and A23187 can stimulate PKM2 dimerization and increase LDH activity, consequently triggering a Warburg effect in human neutrophils (106). In contrast, another study proposed that L-lactate generated during exercise may attenuate PMA-induced NET formation and ROS production (107).

D-lactate demonstrates the capacity to stabilize HIF-1α in a PI3K/Akt pathway-dependent manner and increase the levels of LDHA and PDK-1 in bPMNs (108). Untargeted metabolomic analysis showed that D-lactate amplifies glycolysis and gluconeogenesis in bPMNs. Furthermore, inhibiting glycolysis using 2-DG and 3PO, which are inhibitors of hexokinase and 6-phosphofructo-2-kinase/fructose-2,6-bisphosphatase 3, respectively, resulted in a reduction of NET formation (109) (Figure 3). Additionally, D-lactate stimulates glycogenolysis in bPMNs, which is vital for NET release. This critical attribute was corroborated by the noticeable inhibition observed with CP-91,149, a glycogen phosphorylase inhibitor, emphasizing the significance of glycogen reserves as an energy source pivotal for this mechanism (109).

Mitochondria are the powerhouse of the cell and are responsible of ATP production. The electron transport chain (ETC), located in the inner mitochondrial membrane, consists of four main complexes (I-IV) that sequentially transfer electrons from NADH and FADH_2_ to oxygen to form water (110, 111). As electrons move through these complexes, protons are pumped from the mitochondrial matrix to the intermembrane space by complexes I, III and IV, generating a proton gradient (ΔpH) and an electrical potential (Δψm) (110, 112). Together, these components form the electrochemical potential known as the proton motive force (Δp). The ATP synthase (Complex V) uses the energy from this to cause proton flow back into the matrix to synthesize ATP from ADP and inorganic phosphate (Pi) in a process known as oxidative phosphorylation (111, 113). This mechanism efficiently converts energy from nutrients into ATP, which is the primary energy currency of the cell (111).

Earlier electron microscopy studies usually failed to identify classic mitochondria in neutrophils (114, 115). This suggested that neutrophil mitochondrial function may be primarily confined to apoptotic processes, due to their apparent low mitochondrial count and modest oxidative phosphorylation (OXPHOS) activity (115). However, our previous studies showed that bPMNs possess a functional and complex network of mitochondria (116) with similar characteristics to those described in human PMNs (114). Evidence suggests that mitochondria contribute to the inflammatory process via mitochondrial reactive oxygen species (mtROS) production. This hypothesis is supported by the increase of mtROS in bPMNs by PAF (116). PAF-induced NET release is inhibited by rotenone, a complex I inhibitor (117). Interestingly, mtROS can be generated by complexes I and III of the respiratory chain (118, 119), which serve as the primary source of ROS in neutrophils, particularly in response to NOX-independent stimuli such as calcium ionophores (118).

Interestingly, D-lactate also increases mtROS in bPMNs and its scavenging with mitoTEMPO effectively reduces NET formation (108). In this context, mtROS have been described as key mechanisms for NET release in several diseases and as potential therapeutic targets (120–123). Overactivation of the type I interferon signaling pathway induces ROS and mtROS production, leading to NETosis in the neutrophils of patients with primary Sjögren’s syndrome (124) and a lupus mouse model (123). The mechanism of mtROS induction by D-lactate in blood bPMNs remains unknown; however, it is plausible that it involves metabolic reprogramming with elevation in the expression of hexokinases 2 and 3, as well as phosphofructokinase. This may coincide with the elevated levels of glucose and glucose-6-phosphate detected in the cytosol (109). Lactate can increase glucose metabolism and induce ROS production in chondrocytes via NADPH oxidase 4 (125). Furthermore, preconditioning fibroblasts in a culture medium that relies exclusively on lactate as a fuel source leads to a transition from OXPHOS to glycolysis. This metabolic shift is partly facilitated by the ROS-mediated stabilization of HIF-1α (126). Similarly, mtROS is required for HIF-1α stabilization by hypoxia in human neutrophils (127) and in bPMNs induced by D-lactate under normoxia (108). Moreover, the upregulation of glycolysis in neutrophils promotes enhancements in mtROS production through the glycerol 3-phosphate shuttle (127).

### 4.3 Macrophages

L-lactate can induce lactylation and post-translational modifications (PTMs), which serve as potential mechanisms to regulate inflammation (128). This process involves the inhibition of signaling pathways and the alteration of histones, resulting in the reduction of inflammatory macrophages that drive M2-like polarization towards a reparative phenotype (128, 129). Additionally, evidence also suggests that D-lactate may not directly induce histone lactylation (130). Instead, it can indirectly facilitate the lactylation of cytosolic proteins through S-D-(R)-Lactylglutathione, an intermediate in the glyoxalase pathway. This compound can react non-enzymatically, resulting in lysine D-lactylation (K(D-la)) and PTMs of glycolytic enzymes (131). These differences may explain some of the discrepancies in the response of lactate enantiomers to inflammation observed in bFLS *in vitro* (90). In contrast, D-lactate has been observed to promote histone deacetylase (HDAC) protein gene expression, potentially serving as a crucial transcriptional regulator (132, 133). This association potentially connects the impact of metabolic disturbances to alterations in gene transcription of proinflammatory proteins in cattle. However, further research is required to confirm this hypothesis. Recent findings suggest that D-lactate can induce the transformation of M2 macrophages into M1 macrophages. This modulation would occur by inhibiting the PI3K/Akt pathway while concurrently activating the NF-κB pathway (134). These observations suggest that D-lactate could potentially act as an agonist on TLR2 and TLR9 receptors, increasing the inducible nitric oxide synthase (iNOS), TNF-*α*, and IL-12, thereby exerting influence over the polarization of macrophages towards a pro-inflammatory phenotype (134). TLR2 can activate the PI3K/Akt pathway and NK-κB via MyD88/TAK1 and the expression of pro-inflammatory cytokines (135). Activation of TLR9 induces ERK1/2 and Akt pathways and the IL-6 and TNF-α production in macrophages (136). This finding supports the potential role of D-lactate as a danger-associated metabolite. In contrast, the role of D-lactate in macrophage function could be a more complex scenario. D-lactate exhibits anti-inflammatory effects in experimental models of colitis and endotoxemia, through a specific receptor known as GPR81 (formerly hydroxycarboxylic acid receptor 1, HCAR1) (137). Moreover, D-lactate interfered with M1 polarization provide survival advantage in acute inflammation (137).

### 4.4 T-cells

D-lactate influences T-cell responses by modulating metabolic and signaling pathways that are crucial for their activation, proliferation, and cytokine production (138). Moreover, the interaction of D-lactate with MCTs, particularly MCT-1, highlights its ability to influence T-cell metabolism and function through direct uptake and utilization (138). Unlike L-lactate, which primarily supports glycolysis, D-lactate bypasses cytosolic LDH metabolism, enhances mitochondrial OXPHOS and promote a shift towards energy production through the TCA cycle (139). Additionally, human D-lactate dehydrogenase specifically transfers electrons from D-lactate to cytochrome c, supporting mitochondrial membrane polarization (140). This metabolic shift allows T-cells to sustain prolonged activation and function under conditions of metabolic stress. The TCA cycle and OXPHOS play central roles in regulating T cell-mediated inflammation by providing energy, generating key metabolites, and influencing the differentiation and function of T cell subsets (141). In fact, TCA cycle flux in Th1 and Th17 cells, which controls the elevated succinate/*α*-ketoglutarate (α-KG) ratio, promotes proinflammatory responses by stabilizing HIF-1α and increasing the expression of inflammatory cytokines such as IL-17 (141, 142). Moreover, mitochondrial activity, including the electron transport chain (ETC), generates ROS that act as signaling molecules to modulate T-cell fate and function (142). These metabolic processes are pivotal in maintaining immune homeostasis, and their disruption contributes to the pathogenesis of autoimmune diseases, highlighting potential therapeutic targets for inflammation modulation (141, 142).

D-lactate has been shown to increase mitochondrial ATP production and support pyruvate entry into the TCA cycle, enhancing the energetic capacity of T-cells (143, 144). This metabolic reprogramming favors Th2 polarization, characterized by cytokine production such as IL-4 and IL-13 (138). These cytokines not only modulate inflammatory pathways, but also play a critical role in modulating macrophage polarization from a pro-inflammatory M1 phenotype to an anti-inflammatory M2 phenotype, as discussed previously (145). In addition to its metabolic effects, D-lactate may act as a signaling molecule that synergizes with inflammatory stimuli, such as PMA/ionomycin, to amplify cytokine production in HUT-78 T-cells (138). Notably, this includes its capacity to enhance the expression of Th2 cytokines, which are implicated in the regulation of synovitis and systemic inflammation (145). However, the potential effects of D-lactate on the T-subset of cells could be more complex and require more studies to extend our knowledge of its role in the inflammatory process in cattle.

## 5 Conclusion

Several evidence suggest that D-lactate may enhance glycolytic activity in PMNs, FLS, and macrophages, disrupt cellular function, and that can induce an inflammatory response. Given that an increase in D-lactate is a characteristic of metabolic disorders associated with pathological processes in livestock, including lameness, it may be a relevant factor during the initial steps leading to the inflammatory response. D-lactate may exert pleiotropic effects depending on the affected tissue, as its metabolism relies on LDHD. This implies a more intricate scenario in cells expressing low levels of LDHD, which potentially interferes with mitochondrial function. In addition, the effect of D-lactate on the increase of Th2 cytokine expression may contribute to inflammatory modulation. This aspect warrants further investigation to elucidate the complexities associated with the impact of D-lactate on cellular processes and its potential implications for various pathological conditions in livestock.
